# Pediatric Glioblastoma Multiforme: A Challenging Case of Rapid Growth and Clinical Deterioration in an 11-Year-Old Female Patient

**DOI:** 10.7759/cureus.47697

**Published:** 2023-10-26

**Authors:** Mohammed Khaleel I.KH. Almadhoun, Abd Allah Wasel Hattab

**Affiliations:** 1 Medicine and Surgery, Mutah University, Karak, JOR; 2 Neurosurgery, Mutah University, Karak, JOR

**Keywords:** cns tumors, brain tumors, histopathology and immunohistochemistry, histopathological examination, glioblastoma therapy, pediatric cns tumors

## Abstract

Glioblastoma multiforme (GBM) is an aggressive primary brain tumor that primarily affects adults, with cases in children being extremely rare. Gross total resection with subsequent irradiation and temozolomide, currently delivering the greatest overall survival, is the mainstay of therapy for juvenile GBM. Maximal surgical excision of the visible tumor mass has been shown to have a positive prognostic effect, but radiation concerns for growing brains and inconsistent results from different chemotherapy regimens in pediatric GBM make treatment choices for young patients challenging. Here, we report a case of GBM in an 11-year-old female child who presented with a dramatic presentation of neurologic deficits and clinical worsening due to rapid tumor growth.

## Introduction

Pediatric central nervous system (CNS) tumors are the most prevalent solid tumors in children and the second most common childhood cancer after leukemias. Tumors of glial origin, such as glioblastoma multiforme (GBM), are more common in older patients, whereas tumors of embryonal origin, such as medulloblastoma, are more common in younger children. Of note, only 3-15% of primary CNS tumors in children are GBMs [1,2]. Naturally, this relative rarity has posed a significant barrier to fully unraveling the mystery of juvenile glioblastomas. With a five-year survival rate of less than 20%, the reported median survival in pediatric GBM (p-GBM) ranges from 13 to 73 months [3,4]. Thus, GBM continues to be a severely fatal illness in children with significant morbidity and mortality. Better prognosis and longer survival rates than adults are rarely reported [5]. A patient with GBM typically has a brief clinical history, often less than three months in >50% of patients. Headache, seizure, and cognitive dysfunction are the most frequent presenting signs and symptoms. Focal neurologic impairments and manifestations of elevated intracranial pressure, such as headaches, nausea, and vomiting, are additional presenting symptoms. Despite the fact that most neurosurgeons worldwide follow a similar therapeutic strategy as adults, there is no equivalent standard therapy for children. Gross total resection (GTR) with subsequent irradiation and temozolomide (TMZ), currently delivering the greatest overall survival, is the mainstay of therapy for juvenile GBM patients older than three years of age [6]. Maximal surgical excision of the visible tumor mass has been shown to have a positive prognostic effect, but radiation concerns for growing brains and inconsistent results from different chemotherapy regimens in p-GBM make treatment choices for young patients challenging. Here, we report a case of p-GBM in an 11-year-old female child with a relatively shorter clinical history who unfortunately succumbed to death despite initial improvement with steroids, radiotherapy, and craniotomy.

## Case presentation

An initially healthy 11-year-old girl, displaying typical neuro-psychomotor development, was admitted to the hospital due to a sudden onset of hemiparesis and disorientation that had persisted for 15 days. Left-sided hemiparesis was evident with 3/5 power in the left upper and lower limbs. Her other general and systemic examinations were unremarkable. Her laboratory investigations were within normal limits.

**Figure 1:**
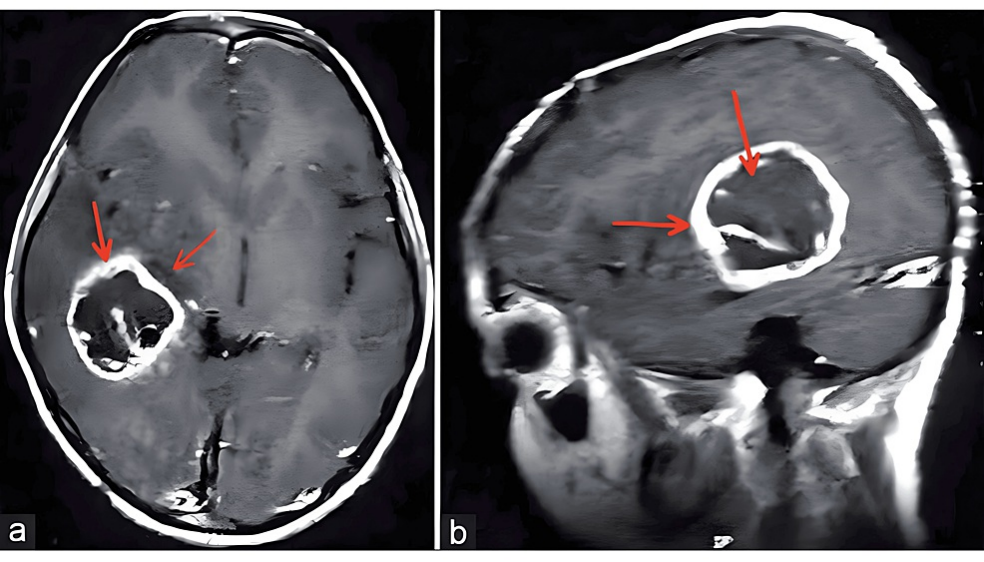
Contrast MRI. Contrast MRI of the brain axial (a) and sagittal images (b) showing a ring-enhancing lesion in the right temporoparietal region with an irregular outline, mass effect, peripheral contrast uptake, and central necrosis.

A skull CT scan revealed an irregularly shaped, deep-seated expansive lesion located on the right side of her brain. The lesion exhibited peripheral contrast enhancement and obstruction to the flow of the cerebrospinal fluid (CSF), as evident in Figure 1. Subsequently, we conducted a ventriculoperitoneal shunt placement and performed a stereotactic biopsy of the lesion.

**Figure 2:**
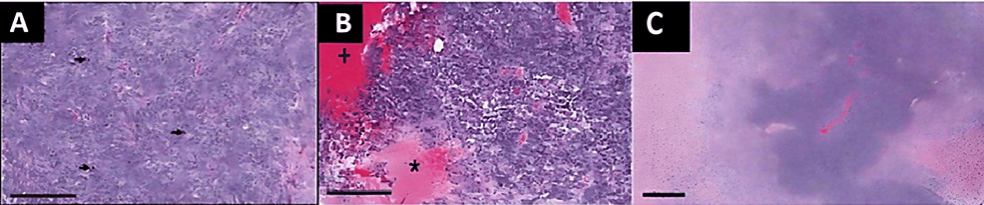
Histological examination. (A-B) Histological examination revealing poorly demarcated, unencapsulated, and invasive neoplasms (C) characterized by marked cell atypia, a high mitotic index (black arrows) (A), and multifocal areas of necrosis (asterisks) and hemorrhage (plus sign) (B). T = tumor, B = normal brain. Scale bar = 200 μm (A-B). Scale bar = 400 μm (C).

Surprisingly, histopathological examination revealed a pleomorphic neoplasm, accompanied by vascular neoformation, and areas of necrosis. The tumor cells displayed elongated morphology with hyperchromatic, pleomorphic nuclei exhibiting atypia and mitotic figures. Immunohistochemistry analysis (Table 1) demonstrated positive staining for glial fibrillary acidic protein (GFAP), Ki-67 proliferation antigen, and S-100 protein. These findings, combined with the observed morphological features and the presence of necrotic tissue, definitively confirmed the diagnosis of GBM (Figure 2).

**Table 1:** Immunohistochemistry. Antigens that were used for the immunohistochemistry.

Antigens	Clone	Result
Glial fibrillary acidic protein	Polyclonal	Positive
Ki-67 cellular proliferation antigen	M1B1	Positive
S-100 protein	Polyclonal	Positive
Neu-N	MAB377	Negative
CD45RB – leukocyte-common antigen (pan-hematopoietic)	PD7/26/16 and 2B11	Negative

**Figure 3:**
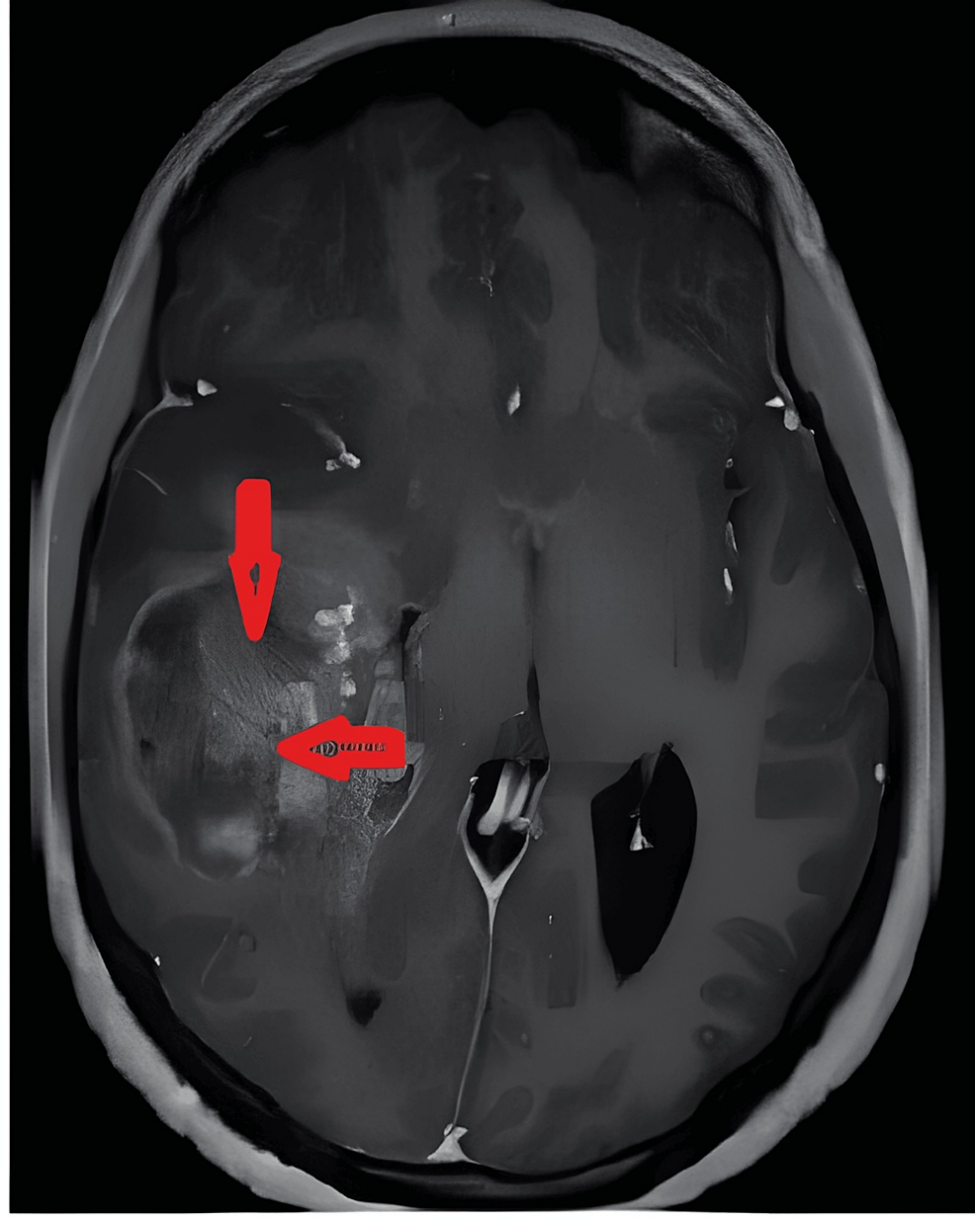
Brain MRI (post-steroid therapy). Treatment MRI (post-steroid therapy) of an 11-year-old girl with right temporal glioblastoma multiforme with heterogeneous enhancement in axial sagittal T1-weighted imaging with contrast agent. The tumor mass had caused a midline shift and showed central enhancement.

The parents were informed of their daughter’s initial diagnosis. They did not expect this diagnosis, so they signed the Against Medical Advice (AMA) paper. We suggested surgical resection of the tumor; however, they denied the procedure. After a month, the girl’s symptoms increased and they returned to our center. Again, this time they did not consent to the surgery, but we at least persuaded the parents to proceed with steroid administration. The patient exhibited improved consciousness levels following steroid administration, but her motor function remained compromised. Consequently, she was referred for radiotherapy. However, after 40 days, a significant neurological deterioration occurred, characterized by hemiplegia and fluctuating levels of consciousness. Subsequent skull CT and MRI scans indicated an enlargement of the lesion (Figure 3). After convincing the parents, we prepared for surgical resection of the tumor. In an effort to alleviate intracranial hypertension, a craniotomy was performed, with partial removal of the tumor. The maximum resection was not possible due to the deep-seated lesion. The postoperative period was uneventful. Tragically, the patient succumbed to her condition 10 days after the surgical procedure.

## Discussion

The WHO classifies GBM as a grade IV glioma, indicating its aggressive nature and resistance to conventional treatments. In p-GBM, different molecular and outcome behaviors are observed. Though rare in children, GBM is an aggressive CNS tumor with a poor prognosis. The majority of GBM patients do not have any known risk factors or a family history of brain tumors. Rarely, tumor predisposition syndromes such as Li-Fraumeni syndrome, Lynch syndrome, or constitutional mismatch repair-deficiency syndrome develop as glioblastomas [7]. The family history in the majority of these cases is notable for having numerous first- and second-degree relatives who develop cancer at an early stage. Typically, a recommendation for genetic counseling is given to such patients. The sole known risk factor for glioblastoma other than genetics is exposure to ionizing radiation, such as that received during therapeutic radiation therapy for leukemia or childhood brain tumors [8]. It can take five years to several decades for a glioma to develop after exposure to radiation.

The location of the tumor and its rate of growth determines the neurological signs and symptoms of glioblastomas. Headache, seizures, and focal neurologic symptoms such as memory loss, motor weakness, visual symptoms, language deficiency, and cognitive and personality changes are the most typical presenting symptoms. The presentation can be dramatic with focal neurologic deficits without common features such as headaches and seizures, as observed in our patient. Children with GBM have acute neurological deterioration often due to intratumoral hemorrhage. This is possible in our patient. Significant edema, mass effect, and elevated intracranial pressure may all be attributed to large tumors. It is well-recognized that preoperative neurological impairments have a deleterious impact on postoperative outcomes [9]. Infants and young children frequently appear with non-specific complaints, such as failure to thrive, lethargy, nausea/emesis, and macrocephaly, which can occasionally be challenging to identify compared to older children.

The majority of p-GBM are supratentorial in location, with cerebral hemispheres accounting for around half of all cases [10]. Some glioblastomas grow very quickly, although the cause of this remains unclear. Rapid symptom worsening may be caused by localized ischemia, tumor growth, or bleeding. Intratumoral hemorrhage can cause glioblastoma to grow as well, which may have occurred in our patient. Intratumoral hemorrhage is hypothesized to occur due to the presence of abnormal tangles of tortuous vessels which get stretched as the tumor grows [11]. Moreover, these vessels are resistant to blood pressure changes making them prone to rupture under stress. Another mechanism of rapid growth of the tumor may be the malignant transformation of initial low-grade glioma to glioblastoma. Although this phenomenon in children is rare (less than 10%), low-grade glioma can expand due to malignant evolution [12].

Neuroimaging is a critical component in GBM diagnosis, therapy, and prognosis. On CT and conventional MRI sequences, these tumors appear as irregular, heterogeneous, contrast-enhancing masses with significant perilesional edema. Necrosis, bleeding, and a garland pattern of enhancement are typically seen in GBMs. The differential diagnosis typically includes disorders such as metastasis, lymphoma, brain abscess, and other illnesses. MRI provides the precise details needed for radiation and surgical planning. For deep-seated cancers that are challenging to biopsy, magnetic resonance spectroscopy frequently reveals a choline peak with reduced N-acetyl aspartate at the tumor site. On diffusion-weighted imaging (DWI), the tumor’s cellular constituents might show constrained diffusion with a low apparent diffusion coefficient. The tumor can frequently be distinguished from a brain abscess using DWI.

In patients with a suspected high-grade glioma such as GBM, a tissue diagnosis is crucial. This can be done either during the surgical resection or during a separate biopsy procedure. When resection is not possible, such as in deep tumors, a significant amount of tumor tissue cannot be removed, the patient’s overall clinical condition prevents surgery, or the patient denies surgery, a biopsy alone is used. Maximal safe resection is the preferred initial strategy for both diagnosis and treatment in the remaining cases.

The best current therapy for adult GBMs involves complete surgical resection followed by chemotherapy and radiation. The same principle is applied to p-GBM. According to the Children’s Cancer Group study, 945 children with high-grade glioma who received surgery to remove 90% or more of the tumor had progression-free (PFS) survival of 35 ± 7% compared to those who did not and had a five-year PFS of 17 ± 4% [13]. However, the tumor’s location and any expansions determine the extent of the tissue resected. In our case, the tumor was deeply seated making it difficult for gross total resection. Tumors in the brainstem, midline supratentorial tumors, tumors affecting the eloquent area, etc., are frequently challenging to entirely remove without causing severe neurological damage. Subtotal resection is an option when gross total resection is not possible because it has been found to increase overall survival and PFS as well [14]. In addition to providing doctors with tissue for diagnosis, surgical debulking (subtotal resection) reduces the mass effect caused by tumors and increases the effectiveness of adjuvant therapy. To assess the degree of resection in patients who undergo surgery, a follow-up brain MRI should be performed 24 to 48 hours after the operation. Post-surgery neuroimaging is a necessary component of the response evaluation criteria, including the McDonald’s and RANO criteria. In this context, neuroimaging is crucial for understanding the real nature of two intriguing radiological events in high-grade gliomas, namely, pseudo-progression and pseudo-response [15].

High-dose glucocorticoids are frequently administered in patients with severe symptoms or herniation risk as they efficiently reduce cerebral edema and ameliorate neurologic deficits. As such chemotherapy regimen is not as well established in p-GBM as in adults. Trials on chemotherapeutic agents such as vincristine, carmustine, procarbazine, hydroxyurea, cisplatin, cytosine arabinoside, prednisone, and dimethyl-triazenoimidazole-carboxamide have not shown any benefit in p-GBM [16]. Although the therapeutic effect of TMZ is well established in adult GBM, the majority of studies show that TMZ chemotherapy has little impact on children’s survival rates except few studies [6]. Radiotherapy doses typically range from 50 to 60 Gy, fractionated over five to six weeks. Due to the severe neurocognitive aftereffects of irradiation and the fact that younger children respond to chemotherapy better than older ones, irradiation is not advised in children less than three years of age. However, the majority of patients still need radiation therapy when they relapse, and the long-term consequences are severe in such young children.

The majority of research highlights poor survival rates in p-GBM compared to adult GBM [3,17], and several indicators are already known to predict survival in p-GBM. In our case, the short duration of symptoms, preoperative neurologic deficits, presence of intense contrast enhancement, and inability to complete gross total tumor resection were associated with poor prognosis.

This case report highlights the possible dramatic clinical presentation, diagnostic workup, and treatment strategies of a rare p-GBM. p-GBM can exhibit rapid growth and consequent rapid neurological deterioration with grave prognosis. This case strongly recommends prompt gross total resection of the tumor without delay. The patients should be persuaded for the same. Moreover, the report aims to contribute valuable insights into the challenges patients face during hospitalization and their subsequent clinical course, thereby enriching existing literature.

## Conclusions

Although rare, p-GBM is different from adult GBM and has a dismal prognosis. p-GBM can potentially present dramatically with much rarer symptoms, and rapid growth of such tumors is probable. The rapid growth of the tumor may be attributable to the tangle of tortuous blood vessel invasion by the aggressive tumor. Prompt surgical intervention with gross total resection of the tumor is warranted in such cases to improve long-term survival. Overall, this case report highlights the clinical presentation, diagnostic workup, and treatment strategies of a rare p-GBM.
